# Spina bifida: a rare clinical image

**DOI:** 10.11604/pamj.2022.42.258.35894

**Published:** 2022-08-08

**Authors:** Kalpesh Gulve, Vaishali Kuchewar

**Affiliations:** 1 Department of Kayachikitsa, Mahatma Gandhi Ayurveda College, Hospital and Research Centre, Salod (Hirapu), Datta Meghe Institute of Medical Sciences (DU), Sawangi, Wardha, India

**Keywords:** Spina bifida, neural tube disease, meningocele

## Image in medicine

Spina bifida is an uncommon neural tube disease (NTD) that affects about one in every 1000 births in Europe. These defects are caused by a teratogenic process that results in the embryonic neural tube's failure to close and aberrant differentiation. Spina bifida occulta, meningocele, myelomeningocele, encephalocele, anencephaly, and dermal sinus are the most common neural tube defects. It occurs within the first 25 days of pregnancy. Although the disease cannot be cured, new medical interventions have allowed many people with spina bifida to live to a ripe old age with a high quality of life. However, long-term monitoring is essential. Women from lower socioeconomic backgrounds are more likely to be affected. A daily intake of folic acid two months prior to conception and two months after conception can prevent approximately 70% of spina bifida. Prenatal diagnosis and terminations of pregnancies, not primary prevention, helped spina bifida become an uncommon condition. Here, we report a case of a one-day-old male neonate who was born at full term and had respiratory distress and a congenital abnormality. A complete local and systemic examination was performed, and meningocele, a severe form of spina bifida, was diagnosed. The patient was referred to a neurosurgeon and was advised an ultrasound abdomen and a magnetic resonance imaging (MRI) spine, but neither of these tests were performed because the patient was transferred to another hospital for treatment.

**Figure 1:**
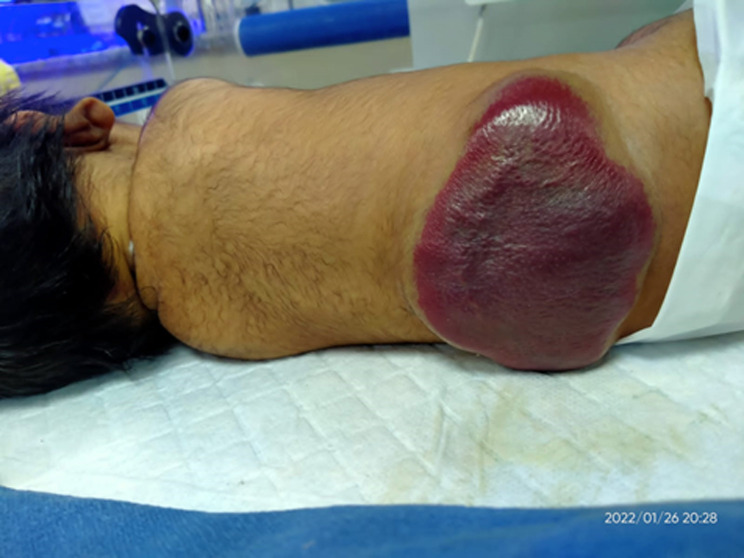
spina bifida

